# Effect of idiopathic epiretinal membrane on macular ganglion cell complex measurement in eyes with glaucoma

**DOI:** 10.3389/fmed.2022.972962

**Published:** 2022-10-26

**Authors:** Shunsuke Nakakura, Ryo Asaoka, Satomi Oogi, Ryota Aoki, Etsuko Terao, Kanae Ueda, Yoshiaki Kiuchi

**Affiliations:** ^1^Department of Ophthalmology, Saneikai Tsukazaki Hospital, Himeji, Japan; ^2^Department of Ophthalmology, Seirei Hamamatsu General Hospital, Hamamatsu, Japan; ^3^Department of Nursing, Seirei Christopher University, Hamamatsu, Japan; ^4^Nanovision Research Division, Research Institute of Electronics, Shizuoka University, Shizuoka, Japan; ^5^The Graduate School for the Creation of New Photonics Industries, Hamamatsu, Japan; ^6^Department of Ophthalmology and Visual Sciences, Graduate School of Biomedical Sciences, Hiroshima University, Hiroshima, Japan

**Keywords:** epiretinal membrane, glaucoma, inner plexiform layer, macular ganglion cell complex, SS-OCT, SUKIMA

## Abstract

**Background/objectives:**

Co-existing idiopathic epiretinal membrane (ERM) and glaucoma complicate the estimation of glaucoma severity via optical coherence tomography (OCT). We investigated the effect of ERM and a new associated parameter, SUKIMA (space between the ERM and retinal surface), on ganglion cell complex (GCC) thickness in eyes with glaucoma, based on a matched comparison of visual field defects.

**Subjects/methods:**

We retrospectively recruited 41 eyes from 34 glaucoma patients with idiopathic ERM and 41 eyes from 41 glaucoma patients without ERM as controls (matched by age, axial length, and mean visual field deviation). The thicknesses of GCC layers [retinal nerve fiber layer (RNFL), ganglion cell layer + inner plexiform layer (GCIPL), and GCC (RNFL + GCIPL)] were measured with swept-source OCT. We investigated the presence of SUKIMA and its effect on GCC measurements.

**Results:**

RNFL, GCIPL, and GCC were thicker in ERM (+) eyes than in control eyes (31.0 ± 12.3 μm vs. 22.7 ± 10.8 μm, 62.6 ± 12.2 μm vs. 53.8 ± 5.9 μm, and 91.8 ± 16.6 μm vs. 76.8 ± 13.3 μm, respectively; *P* < 0.01). Eyes in the ERM-associated SUKIMA (+) group had thicker GCIPL and GCC than those in the ERM-associated SUKIMA (−) and control groups (*P* < 0.01).

**Conclusion:**

ERM-associated SUKIMA affects GCC thickness and can result in underestimations of glaucoma severity. We should check for the presence of ERM using a B mode scan as well as check for the SKIMA sign.

## Introduction

Glaucoma is currently the leading cause of irreversible blindness in the world (1), and the number of newly diagnosed cases is increasing due to increased life expectancy (2). Optical coherence tomography (OCT), including the measurements of circumpapillary retinal nerve fiber layer (cpRNFL) thickness and macular ganglion cell complex (GCC) [RNFL + ganglion cell layer (GCL) + inner plexiform layer (IPL)], is the primary diagnostic method for glaucoma (3–5). Idiopathic epiretinal membrane (ERM) is a common retinal disease that is also diagnosed using OCT (6). Similar to glaucoma, the prevalence of ERM increases with age (7). Furthermore, cataract surgery increases ERM (8). The comorbidity of glaucoma and ERM can affect OCT measurements (9, 10). Specifically, eyes with ERM have been reported to have thicker cpRNFL (11–13) and GCC (14) than those of normal eyes (15). Among glaucoma patients, the effect of ERM on retinal structures remains unknown. Sakimoto et al. reported that glaucoma eyes with ERM had worse visual fields than those without (16). However, the mechanism by which ERM changes the retinal structure, especially in critical GCC layers, among those with similarly damaged visual fields has not yet been clarified.

Recently, Murase et al. defined and measured a novel OCT parameter, termed SUKIMA, as the gap between the ERM and retinal surface (17). We hypothesized that SUKIMA, which is concerned with metamorphopsia, could affect GCC layer thickness and OCT glaucoma measurements. Our study aimed to investigate the effect of ERM and the associated SUKIMA on GCC layer thickness in glaucoma by comparing visual field damage, age, and axial length between glaucoma eyes with ERM and matched-control glaucoma eyes.

## Materials and methods

This retrospective cross-sectional comparative study was approved by the Institutional Review Board of Saneikai Tsukazaki Hospital (IRB No. 211020) and performed in accordance with the tenets of the Declaration of Helsinki. Information in the electronic database of the Department of Ophthalmology, Saneikai Tsukazaki Hospital, was collected between June 2018 and May 2021.

### Patients

Patients were included if they had glaucoma or suspected glaucoma (ocular hypertension or pre-perimetric glaucoma) with ERM as diagnosed via a glaucomatous optic disk and visual field test using Humphrey Field Analyzer 24-2 test according to the Swedish Interactive Threshold Algorithm fast 24-2 program (HFA 24-2, Carl Zeiss Meditec, Dublin, CA). ERM was observed using radial B-scan of swept-source (SS)-OCT. Patients were excluded if they were below 20 years old or had a history of glaucoma surgery or any other retinal disease besides glaucoma or ERM. All patients had documented records of the HFA central 10-2 test, axial length with IOLMaster 700 (Carl Zeiss Meditec, Jena, Germany), and SS-OCT (DRI OCT Triton, Topcon Corp., Japan). A reliable visual field test was defined as < 33% response of fixation losses, false-positive responses, and false-negative responses.

In addition, we retrospectively enrolled glaucoma patients without ERM as controls via a one-to-one correspondence with glaucoma patients with ERM based on the following inclusion criteria: (1) age (± 5 years old) and (2) axial length (± 1 mm) because GCC thickness is affected by age and axial length (18) as well as (3) a mean deviation in HFA central 10-2 test of ± 2 dB.

The inclusion and exclusion criteria for controls were similar to those of patients with glaucoma or suspected glaucoma with ERM. Visual acuity and intraocular pressure, measured using a Goldmann applanation tonometer, were used for testing HFA central 10-2.

### Ganglion cell complex analysis

**FIGURE 1:**
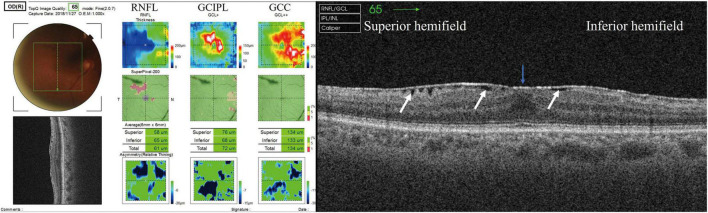
GCC mapping (RNFL, GCIPL, and GCC) and radial scan. **(Left panel)** GCC thickness map obtained using SS-OCT. RNFL thickness + GCIPL thickness = GCC thickness. **(Right panel)** White arrow showing SUKIMA. Severity of ERM was grade 2 and SUKIMA was observed in both superior and inferior hemifields.

SS-OCT (DRI OCT Triton, Topcon Corp., Japan) was performed at a center wavelength of 1,050 nm and a scan speed of 100,000 axial scans per second. Eyes were imaged using the three-dimensional scan mode, with an A-scan density of 512 lines (horizontal) × 128 lines (vertical). Super Pixel-200 was used to obtain macular 7.0 mm × 7.0 mm scans. The 6.0 mm × 6.0 mm macular thickness layer analyses were displayed as RNFL; GCL + IPL (GCIPL) was displayed as GCC +; and GCC (RNFL + GCIPL) was displayed as GCC + + (Figure 1, left panel). Only measurements with quality scores > 40 were included in this study.

All GCC thickness values were displayed as the superior hemifield (3.0 mm × 6.0 mm), inferior hemifield (3.0 mm × 6.0 mm), and total average (6.0 mm × 6.0 mm (Figure 1, left panel). The presence and degree of ERM were evaluated using a radial B-scan across the fovea, and we classified the degree of ERM using Govetto’s grading system as follows (19): Stage 1, presence of the foveal pit and well-defined retinal layers; Stage 2, absence of the foveal pit and well-defined retinal layers; Stage 3, absence of the foveal pit, well-defined retinal layers, and presence of ectopic inner foveal layer; and Stage 4, absence of the foveal pit, disrupted retinal layers, and presence of ectopic inner foveal layers. If apparent segmentation error was observed, we manually modified each segmentation line in the retina.

We evaluated the presence or absence of a gap between the ERM and retinal surface (SUKIMA), an OCT parameter found in patients with ERM as reported by Murase et al., that may affect visual acuity and metamorphopsia (17) (Figure 1, right panel). All the OCT parameters and the presence of SUKIMA were evaluated at the superior hemifield, inferior hemifield, and total field, along with the average HFA 10-2 total deviation (TD) in the corresponding area.

The presence/absence of SUKIMA and ERM was ascertained by S.N. and S.O. and included in the current study only when there was an agreement between the two graders.

### Statistical analysis

Statistical analyses were performed using BellCurve for Excel (Social Survey Research Information Co., Tokyo, Japan) and R software (version 3.6.1).^1^ The Shapiro–Wilk test was used to verify normal distribution. For the comparison of patients’ backgrounds, the X^2^-test and Welch’s *t*-test (two-tailed) were used. The best-corrected visual acuity (logMAR) was compared using Welch’s t-test. RNFL, GCPIL, and GCC were compared among the SUKIMA (+), SUKIMA (−), and control groups using the Tukey test after adjusting for age, sex, axial length, and HFA 10-2 TD of the relevant area. In total field evaluation, the patient was considered SUKIMA (+) when SUKIMA was observed in either the superior or inferior fields.

The association between the grade of ERM (19) and presence of SUKIMA (17) was investigated for each of the superior and inferior hemifields using mixed-effects logistic regression analysis, where random effects were subjects and eyes.

We determined the association between ERM degree [according to Govetto’s grading system (stages 1–4)] (19) and RNFL and GCIPL thickness using linear regression after adjusting for age, sex, axial length, and TD of the relevant area via the Tukey test.

*P*-values of < 0.05 were considered statistically significant. The sample size was estimated to be 41 patients for detecting a 10.3-mm difference between the groups, with a significance level of 5% and power of 80% according to an SD of 16.6 mm for GCC thickness in ERM (+) glaucoma.

## Results

We enrolled 41 eyes of 34 glaucoma patients with ERM and 41 eyes of 41 control glaucoma patients without ERM. Patients’ backgrounds are shown in Table 1. We found no significant difference in sex, age, glaucoma type, axial length, intraocular pressure, and visual acuity between the two groups. The HFA parameters, mean deviation, HFA 10-2 TD superior hemifield, HFA 10-2 TD inferior hemifield, and foveal threshold were similar between the two groups (*P* > 0.05). However, macular thickness measurements, mean RNFL, GCIPL, and GCC were thicker in ERM (+) eyes than in control eyes (*P* < 0.05; Table 1).

**TABLE 1:** Patient demographic, HFA, and OCT data.

	ERM(+) glaucoma	ERM(−) glaucoma	*P-value*
Number of eyes/patients	41/34	41/41	
Sex (female),%	21 (61)	19 (46)	0.182
Median age (quantile)	69 (63, 76)	68 (63, 76)	0.818
Glaucoma type			0.368
POAG (n,%)	38 (93)	32 (78)	
EFG (n,%)	0	1 (2)	
SG (n,%)	0	2 (5)	
Combined glaucoma (n,%)	0	2 (5)	
ACG (n,%)	2 (5)	2 (5)	
OH (n,%)	0	1 (2)	
PPG (n,%)	1 (2)	1 (2)	
Axial length (mm)	25.2 ± 2.0	25.1 ± 1.9	0.684
IOP (mmHg)	14.0 ± 2.6	14.2 ± 1.9	0.707
HFA 24-2			
Mean deviation (dB)	−12.0 ± 8.6	−11.6 ± 8.7	0.815
Pattern standard deviation(dB)	9.0 ± 4.6	8.6 ± 3.9	0.713
HFA central 10-2			
Mean deviation (dB)	−13.4 ± 8.8	−12.9 ± 8.6	0.761
TD superior field (dB)	−18.1 ± 11.8	−15.8 ± 10.3	0.346
TD inferior field (dB)	−9.1 ± 9.8	−10.2 ± 10.0	0.620
TD total field (dB)	−13.6 ± 8.9	−13.0 ± 8.5	0.748
Foveal threshold (dB)	31.8 ± 6.6	33.0 ± 3.4	0.330
Visual acuity (logmar)	0.14 ± 0.15	0.13 ± 0.19	0.868
Macular GCC layer thickness			
RNFL superior field (μm)	34.4 ± 11.6	26.2 ± 12.6	0.003
RNFL inferior field (μm)	25.7 ± 13.3	19.4 ± 10.8	0.019
RNFL total field (μm)	31.0 ± 12.3	22.7 ± 10.8	0.001
GCIPL superior field (μm)	64.3 ± 14.0	54.7 ± 7.4	0.000
GCIPL inferior field (μm)	59.0 ± 9.4	53.2 ± 5.9	0.001
GCIPL total field (μm)	62.6 ± 12.2	53.8 ± 5.9	0.000
GCC superior field (μm)	98.5 ± 20.8	80.9 ± 17.1	0.000
GCC inferior field (μm)	84.8 ± 16.3	71.3 ± 12.1	0.000
GCC total field (μm)	91.8 ± 16.6	76.8 ± 13.3	0.000
SKIMA (+) superior field (N,%)	19 (46)	–	–
SKIMA (+) inferior field (N,%)	13 (31)	–	–
ERM grading using Govetto et al. (19)		–	–
Stage 1 (n,%)	20 (49)	–	–
Stage 2 (n,%)	17 (41)	–	–
Stage 3 (n,%)	3 (7)	–	–
Stage 4 (n,%)	1 (2)	–	–

Data are expressed as mean ± standard deviation. POAG, primary open angle glaucoma; EFG, exfoliation glaucoma; SG, secondary glaucoma; OH, ocular hypertension; ACG, angle closure glaucoma; PPG, pre-perimetric glaucoma; IOP, intraocular pressure. *P*-values were provided using X^2^-test or Welch’s *t*-test.

### Effect of SUKIMA on ganglion cell complex thickness

The ERM grade corresponded with the presence of SUKIMA at the superior and inferior hemifield (*p* = 0.019, by mixed-effects logistic regression) (Supplementary Digital Content 1).

**FIGURE 2:**
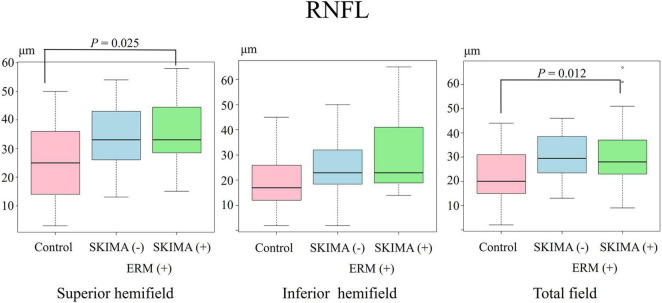
Effect of SUKIMA on RNFL thickness. Superior hemifield **(Left panel)** significant difference was found between the SUKIMA (+) and control groups. Inferior hemifield **(Middle panel)** no significant difference was found among the groups. Total field **(Right panel)** significant difference was observed between the SUKIMA (+) and control groups.

**FIGURE 3:**
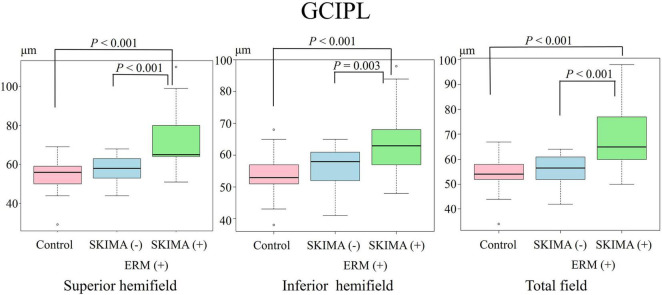
Effect of SUKIMA on GCIPL thickness. Superior hemifield **(Left panel)** significant difference was found between the SUKIMA (+) and control groups and between the SUKIMA (+) and SUKIMA (−) groups. Inferior hemifield **(Middle panel)** significant difference was found between the SUKIMA (+) and control groups and between the SUKIMA (+) and SUKIMA (−) groups. Total field **(Right panel)** significant difference was found between the SUKIMA (+) and control groups and between the SUKIMA (+) and SUKIMA (−) groups.

**FIGURE 4:**
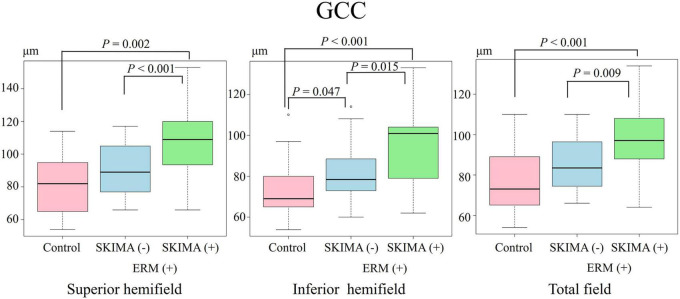
Effect of SUKIMA on GCC thickness. Superior hemifield **(Left panel)** significant difference was found between SUKIMA (+) and control groups and between SUKIMA (+) and SUKIMA (−) groups. Inferior hemifield **(Middle panel)** significant difference was found between each group. Total field **(Right panel)** significant difference was found between the SUKIMA (+) and control groups and between the SUKIMA (+) and SUKIMA (−) groups.

Patients’ backgrounds among the SKIMA (+), SKIMA (−), and control groups are shown in Supplementary Digital Content 2. There was no significant difference among the groups in the inferior hemifield and total field comparisons, except for in HFA 10-2 TD superior field (dB) (*P* = 0.006, by ANOVA) of the superior hemifield. The results of comparison of each field using the Tukey test adjusted for age, sex, axial length, and HFA 10-2 TD of the relevant area are shown in Figures 2–4.

There was a significant difference in the superior hemifield RNFL thickness between the SUKIMA (+) and control groups (35.1 ± 12.3 μm versus 26.2 ± 12.2 μm; P = 0.025; Figure 2, left panel). The total mean RNFL thickness was also significantly higher in the SUKIMA (+) group than in the control group (31.7 ± 14.7 μm versus 22.7 ± 10.8 μm; *P* = 0.012; Figure 2, right panel). In contrast, in the inferior hemifield, the difference in RNFL thickness was insignificant among the three groups (*P* > 0.05; Figure 2, middle panel).

There was also a significant difference in the mean GCIPL thickness between the SUKIMA (+) and control groups (73.1 ± 15.2 μm versus 54.7 ± 7.4 μm; *P* < 0.001) and between the SUKIMA (+) and SUKIMA (−) groups (73.1 ± 15.2 μm versus 56.7 ± 6.8 μm; *P* < 0.001; Figure 3, left panel). Moreover, there was a significant difference in the mean GCIPL thickness in the inferior hemifield between the SUKIMA (+) and control groups (65.2 ± 11.3 μm versus 53.2 ± 5.9 μm; *P* < 0.001) and between the SUKIMA (+) and SUKIMA (−) group (65.2 ± 11 μm versus 56.1 ± 6.6 μm; *P* = 0.003; Figure 3, middle panel. Another significant difference was noted between the SUKIMA (+) and control groups (69.4 ± 12.6 μm versus 53.8 ± 5.9 μm; *P* < 0.001) and between the SUKIMA (+) and SUKIMA (−) groups (69.4 ± 12.6 μm versus 55.5 ± 6.2 μm; *P* < 0.001; Figure 3, right panel).

On the other hand, the mean GCC thickness in the superior hemifield was significantly higher in the SUKIMA (+) group than in the control group (108.0 ± 23 μm versus 80.9 ± 17.1 μm; *P* = 0.002), and a similar significant difference was noted between the SUKIMA (+) and SUKIMA (−) groups (108.0 ± 23 μm versus 90.3 ± 14.9 μm; *P* < 0.001; Figure 4, left panel).

There was a significant difference in the mean GCC thickness in the inferior hemifield between the SUKIMA (+) and control groups (94.3 ± 19.2 μm versus 72.3 ± 12.1 μm; *P* < 0.001), between the SUKIMA (+) and SUKIMA (−) groups (94.3 ± 19.2 μm versus 80.3 ± 12 μm; *P* = 0.015), and between the SUKIMA (−) and control groups (80.3 ± 12 μm versus 72.3 ± 12.1 μm; *P* = 0.047; Figure 4, middle panel). The mean GCC thicknesses in the total hemifield in the SUKIMA (+), SUKIMA (−), and control groups were 97.5 ± 17.6 μm (*N* = 21), 85.8 ± 13.5 μm (*N* = 20), and 76.8 ± 13.3 μm (*N* = 41), respectively. There was a significant difference in the total mean GCC thickness between the SUKIMA (+) and control groups (97.5 ± 17.6 μm versus 76.8 ± 13.3 μm; *P* < 0.001) and between the SUKIMA (+) and SUKIMA (−) groups (97.5 ± 17.6 μm versus 85.8 ± 13.5 μm; *P* = 0.009; Figure 4, right panel).

### Effect of epiretinal membrane stage on retinal nerve fiber layer and GCIPL thickness

We found no significant difference between ERM stage and RNFL thickness; however, there was a significant tendency between ERM stage and GCIPL, particularly in GCC (Supplementary Digital Content 3).

## Discussion

In this study, glaucoma eyes with ERM had thicker GCCs than those without ERM, despite similar visual field disturbances. Thus, the severity of glaucoma may be underestimated when a GCC map is used without checking for the presence of ERM using the B mode scan in SS-OCT. Our findings also indicate that glaucoma severity may be highly underestimated in the presence of SUKIMA.

Eyes with glaucoma and ERM-associated SUKIMA (+) had significantly thicker GCIPL and GCC layers than those in SUKIMA (−) or control eyes. We consider that presence of SUKIMA and the severity of ERM, as classified by Govetto et al. (19), are related. Thus, the advanced stage of ERM will more likely present excess retinal layer thickness due to the presence of SUKIMA. Additionally, Govetto et al. reported that the inner retinal layers of the macula may be especially sensitive to tractional stress, and ERM formation may significantly alter the inner foveal microanatomy (19). Further, Lee et al. reported that an increase in cpRNFL thickness was positively correlated with ERM severity, unrelated to software segmentation error (12). Other previous studies have reported that the visual outcome of ERM surgery was predominantly dependent on the retinal outer layer structures, such as inner segment/outer segment junction (20, 21), although the reduction in thicknesses of GCIPL (22), GCC (14, 23), inner nuclear layer (23), and outer nuclear layer (23) were also influential. During the structural and functional follow-up in patients with glaucoma, Rabiolo et al. reported that in eyes with 10-2 visual field worsening, GCC and GCL demonstrated the fastest rates of change among full macular thickness and GCC, GCIPL, GCL, and outer retinal layer thicknesses (24).

In short, our results suggest that thickening of the retinal layer and disruptions to structural homeostasis should be carefully monitored in eyes with ERM. Additionally, Mohammadzadeh et al. reported that GCC is the optimal macular measurement structure for the detection of structural changes in eyes with moderate to severe glaucoma (25). Therefore, measuring the true thickness of the GCC layer is critical for monitoring glaucoma progression. The comorbidity of ERM and glaucoma increases the difficulty of using GCC to monitor glaucoma, whereas ERM removal has been shown to increase visual functional deterioration (26, 27).

The comorbidity of ERM and glaucoma is problematic not only for the diagnosis and monitoring of glaucoma but also for its pathology. For instance, the tractional force of ERM can change retinal layer thicknesses, causing foveal pit disappearance, and can disrupt retinal structures (6, 13, 19).

There were several limitations in this study. First, only the presence or absence of SUKIMA was determined from the radial OCT scans. In the study by Murase et al. (17), which defined and calculated SUKIMA from vertical and horizontal OCT images, the investigators quantitatively calculated the space of SUKIMA and showed a relationship between size and metamorphopsia scores. Thus, SUKIMA size measurements may provide additional information regarding the impact of ERM. Second, this was a cross-sectional rather than a longitudinal study; thus, we were unable to identify the relationship between the progression of ERM severity and that of glaucoma.

## Conclusion

Glaucoma eyes with ERM have thicker GCC layers than those without ERM, despite similar visual field disturbance. The presence of ERM as well as associated SUKIMA should be checked for when assessing the GCC thickness to limit underestimating the severity of glaucoma. We should check for the presence of ERM using a B mode scan as well as check for the SKIMA sign.

## Precis

Especially, glaucoma eyes with an epiretinal membrane (ERM) associated SUKIMA have thicker ganglion cell complex layers than those without ERM despite similar visual field defects.

## Data availability statement

The original contributions presented in this study are included in the article/Supplementary material, further inquiries can be directed to the corresponding author/s.

## Ethics statement

The studies involving human participants were reviewed and approved by Institutional Review Board of Saneikai Tsukazaki Hospital (IRB No. 211020). The patients/participants provided their written informed consent to participate in this study.

## Author contributions

SN, RAs, and YK designed and supervised the study. SN and RAs analyzed, interpreted the data, and drafted the manuscript. All authors were responsible for data acquisition, contributed to refinement of the study protocol, and approved the final manuscript.

## Footnotes

1
http://www.rproject.org/
