# Supplementary material for: Effect of idiopathic epiretinal membrane on macular ganglion cell complex measurement in eyes with glaucoma
Source: Front Med (Lausanne). 2022 Oct 26;9:972962. doi: 10.3389/fmed.2022.972962 (PMC9644160; doi:10.3389/fmed.2022.972962)
Supplement: Supplementary file 1 [file Table_1.docx]

Supplemental Digital Content 1. Presence of SUKIMA and epiretinal membrane severity

|  | Superior hemifield | |  | Inferior hemifield | |  | Total field | |
| --- | --- | --- | --- | --- | --- | --- | --- | --- |
|  | SUKIMA (+) | SUKIMA (−) |  | SUKIMA (+) | SUKIMA (−) |  | SUKIMA (+) | SUKIMA (−) |
| Stage 1 | 4 | 16 |  | 4 | 16 |  | 6 | 14 |
| Stage 2 | 12 | 5 |  | 7 | 10 |  | 12 | 5 |
| Stage 3 | 2 | 1 |  | 1 | 2 |  | 2 | 1 |
| Stage 4 | 1 | 0 |  | 1 | 0 |  | 1 | 0 |

ERM staging according to Govetto’s grading system (19)
