# Supplementary material for: Effect of idiopathic epiretinal membrane on macular ganglion cell complex measurement in eyes with glaucoma
Source: Front Med (Lausanne). 2022 Oct 26;9:972962. doi: 10.3389/fmed.2022.972962 (PMC9644160; doi:10.3389/fmed.2022.972962)
Supplement: Supplementary file 2 [file Table_2.docx]

Supplemental Digital Content 2. Comparison of background data of patients with/without the SKIMA sign with that of the controls

|  | Superior hemifield | | | |  | Inferior hemifield | | | |  | Total field | | | |
| --- | --- | --- | --- | --- | --- | --- | --- | --- | --- | --- | --- | --- | --- | --- |
|  | ERM (+) SKIMA (+) | ERM (+) SKIMA (−) | Control | P value |  | ERM (+) SKIMA (+) | ERM (+) SKIMA (−) | Control | P value |  | ERM (+) SKIMA (+) | ERM (+) SKIMA (−) | Control | P value |
| Number of eyes/patients | 19/15 | 22/19 | 41/41 |  |  | 13/11 | 28/23 | 41/41 |  |  | 21/18 | 20/16 | 41/41 |  |
| Sex (female), % | 9 (50) | 13 (65) | 19 (46) | 0.385 |  | 6 (54) | 16 (66) | 19 (46) | 0.347 |  | 10 (61) | 12 (46) | 19 (46) | 0.148 |
| Median age (quantile) | 69 (64, 75) | 69 (62, 77) | 68 (63, 76) | 0.925 |  | 69 (63, 75) | 69 (64, 77) | 68 (63, 76) | 0.861 |  | 67 (64, 75) | 69 (63, 76) | 68 (63, 76) | 0.945 |
| Glaucoma type |  |  |  | 0.674 |  |  |  |  | 0.783 |  |  |  |  | 0.621 |
| POAG (n, %) | 19 (100) | 19 (86) | 32 (78) |  |  | 13 (100) | 25 (89) | 32 (78) |  |  | 21 (100) | 17 (85) | 32 (78) |  |
| PXG (n, %) | 0 | 0 | 1 (2) |  |  | 0 | 0 | 1 (2) |  |  | 0 | 0 | 1 (2) |  |
| SG (n, %) | 0 | 0 | 2 (5) |  |  | 0 | 0 | 2 (5) |  |  | 0 | 0 | 2 (5) |  |
| Combined glaucoma (n, %) | 0 | 0 | 2 (5) |  |  | 0 | 0 | 2 (5) |  |  | 0 | 0 | 2 (5) |  |
| ACG (n, %) | 0 | 2 (9) | 2 (5) |  |  | 0 | 2 (7) | 2 (5) |  |  | 0 | 2 (10) | 2 (5) |  |
| OH (n, %) | 0 | 0 | 1 (2) |  |  | 0 | 0 | 1 (2) |  |  | 0 | 0 | 1 (2) |  |
| PPG (n, %) | 0 | 1 (5) | 1 (2) |  |  | 0 | 1 (4) | 1 (2) |  |  | 0 | 1 (5) | 1 (2) |  |
| Axial length (mm) | 25.0±2.0 | 25.4±2.0 | 25.1±1.9 | 0.733 |  | 25.2±1.6 | 25.3±2.2 | 25.1±1.9 | 0.920 |  | 25.2±2.0 | 25.4±2.0 | 25.1±1.9 | 0.874 |
| IOP (mmHg) | 14.2±3.0 | 13.8±2.3 | 14.2±1.9 | 0.805 |  | 14.4±2.4 | 13.8±2.7 | 14.2±1.9 | 0.695 |  | 14.2±2.9 | 13.8±2.4 | 14.2±1.9 | 0.811 |
| HFA central 10-2 |  |  |  |  |  |  |  |  |  |  |  |  |  |  |
| Mean deviation (dB) | −15.5±8.8 | −11.6±8.7 | −12.9±8.6 | 0.349 |  | −13.2±9.6 | −13.5±8.6 | −12.9±8.6 | 0.964 |  | −14.8±8.8 | −12.0±8.9 | −12.9±8.6 | 0.578 |
| TD upper field (dB) | −23.7±10.2 | −13.3±11.2 | −15.8±10.3 | 0.006 |  | −19.0±12.7 | −17.7±11.6 | −15.8±10.3 | 0.607 |  | −21.7±11.5 | −14.4±11.2 | −15.8±10.3 | 0.068 |
| TD lower field (dB) | −8.2±10.5 | −10.0±9.3 | −10.2±10.0 | 0.758 |  | −7.9±11.8 | −9.7±8.9 | −10.2±10.0 | 0.757 |  | −8.6±10.5 | −9.7±9.3 | −10.2±10.0 | 0.828 |
| TD total (dB) | −15.9±8.8 | −11.6±8.7 | −13.0±8.5 | 0.275 |  | −13.4±9.7 | −13.7±8.7 | −13.0±8.5 | 0.966 |  | −15.1±8.8 | −12.1±8.9 | −13.0±8.5 | 0.506 |
| Foveal threshold (dB) | 31.4±6.5 | 32.2±6.8 | 33.0±3.4 | 0.554 |  | 32.1±7.2 | 31.7±6.4 | 33.0±3.4 | 0.605 |  | 31.6±6.2 | 32.0±7.1 | 33.0±3.4 | 0.607 |
| Visual acuity (logmar) | 0.15±0.20 | 0.12±0.11 | 0.13±0.19 | 0.874 |  | 0.14±0.24 | 0.14±0.10 | 0.13±0.19 | 0.986 |  | 0.14±0.19 | 0.13±0.11 | 0.13±0.19 | 0.974 |

P values were calculated using the X^2^ test or ANOVA among groups.
