# Supplementary material for: Effect of idiopathic epiretinal membrane on macular ganglion cell complex measurement in eyes with glaucoma
Source: Front Med (Lausanne). 2022 Oct 26;9:972962. doi: 10.3389/fmed.2022.972962 (PMC9644160; doi:10.3389/fmed.2022.972962)
Supplement: Supplementary file 3 [file Table_3.docx]

Supplemental Digital Content 3. Relation between ERM stage and RNFL or GCIPL thickness.


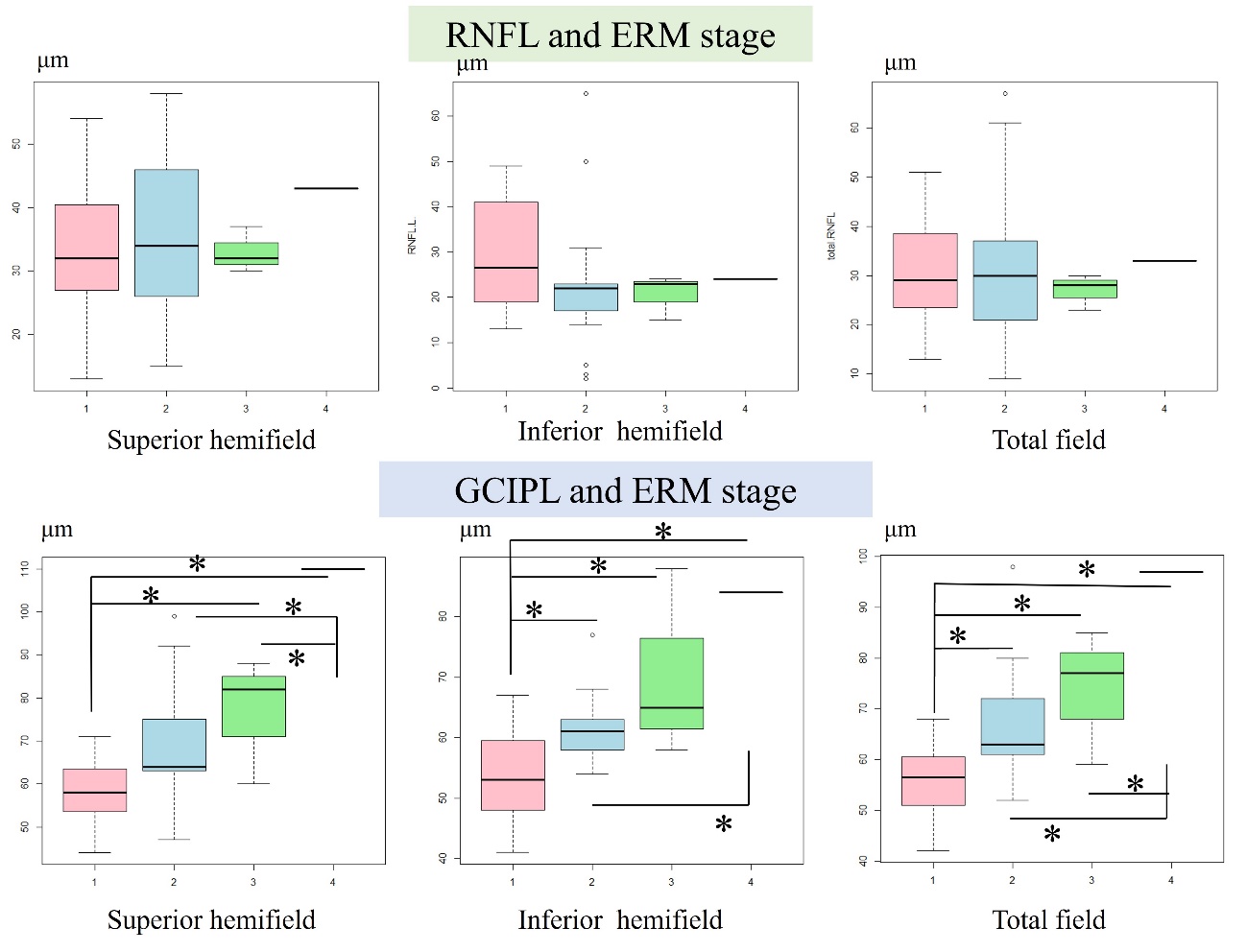


Asterisks indicate pairs with significant differences (P <0.05), as determined using Tukey multiple comparison tests. There was no apparent tendency between RNFL thickness and the stage of ERM degree. However, there was a tendency between increasing GCIPL thickness and ERM stage. GCC = RNFL + GCIPL; therefore, GCC shows similar tendency as that of GCIPL.
